# Euthanasia of Danish dairy cows evaluated in two questionnaire surveys

**DOI:** 10.1186/1751-0147-50-33

**Published:** 2008-08-21

**Authors:** Peter T Thomsen, Jan Tind Sørensen

**Affiliations:** 1 University of Aarhus, Faculty of Agricultural Sciences, Department of Animal Health, Welfare and Nutrition, P. O. Box 50, DK-8830 Tjele, Denmark

## Abstract

**Background:**

Mortality risk in Danish dairy cows has more than doubled since 1990 (from 2% in 1990 to 5% in 2005). Until now, registrations about dead cows in the Danish Cattle Database have not included information about whether the cow died unassisted or was euthanized.

**Methods:**

We interviewed a random sample of 196 Danish dairy farmers that had reported a dead cow to the Danish Cattle Database in 2002 and 196 dairy farmers that had reported a dead cow in 2006. Our objectives were to evaluate the proportion of euthanized cows, changes in the behaviour of farmers regarding euthanasia of cows over the years and possible reasons for these changes.

**Results:**

It seems that the threshold for euthanasia of cows among farmers has changed. Farmers generally reported a lower threshold for euthanasia compared to 5–10 years ago.

**Conclusion:**

The threshold for euthanasia of cows has, according to the dairy farmers, become lower. This might have positive impacts on animal welfare as more seriously ill cows are euthanized in the herds and not put through a period of suffering associated with disease and treatment or transported to a slaughterhouse in poor condition.

## Background

The peer reviewed literature on dairy cow mortality is relatively sparse. In a review on dairy cow mortality, Thomsen and Houe [1] concluded that the number of published studies is surprisingly low, especially seen in relation to the large impact of dairy cow mortality on animal welfare and the farmer's economy.

Mortality risk defined as unassisted death and euthanasia in Danish dairy cows has increased significantly since 1990. The mortality risk has increased from approximately 2% in 1990 to 5% in 2005. This increase is seen for all major dairy breeds and for all parities. There has been only a slight increase in mortality risk during the period 2002 to 2005 (from approximately 4.7% to 4.9%). Throughout the years the mortality risk has been approximately twice as high in older cows (parity 3 or older) as in younger cows [2,3]. At first glance, this development seems very negative, as mortality risk in Danish dairy cows has more than doubled since 1990. The increased mortality can be caused by an increasing number of cows dying unassisted or by an increasing number of euthanized cows (or both). Cows dying unassisted probably often constitute an animal welfare problem, as cows dying unassisted in many cases will suffer from fear or pain before death. The situation concerning euthanasia is, however, more complex. An increase in the number of euthanized cows might be due to an increased number of seriously ill cows. This situation also has negative impacts on animal welfare. If, on the other hand, an increase in the number of euthanized cows is not a consequence of increased morbidity, but caused by an altered threshold for euthanasia of cows among farmers, it might have a positive impact on animal welfare. More seriously ill cows might be euthanized at an early stage and thus not put through a (perhaps long) period of suffering associated with disease and treatment attempts.

The objectives of this study were to examine the proportion of dead Danish dairy cows that had been euthanized in 2002 and 2006, to examine the development over time in the threshold for euthanasia of cows based on interviews with farmers, to evaluate the farmers' perceptions regarding the background of this development and finally to analyse the welfare implications.

## Methods

In Denmark, farmers are required by law to report all deaths in cows to the Danish Cattle Database. Registration is based on the mandatory identification by ear tags and a central computerized tracking system. Consequently, a registration rate very close to 100% is achieved. Until now, these registrations have not distinguished between cows dying unassisted and cows being euthanized on the farm. They were all simply recorded as 'dead'. Until recently, it was therefore unknown how many cows died unassisted and how many were euthanized and whether there has been a change in the prevalence of euthanized cows over the years. Over the course of 7 weeks in 2002, and again in 2006, we randomly identified four cows on a daily basis that had been reported as 'dead' to the Danish Cattle Database. The random samples in each of the two years were independent. Dead cows included unassisted dead and euthanized cows, but not cows slaughtered. As all Danish dairy farmers are required by law to report dead cows to the Danish Cattle Database, the sample population for the study was all Danish dairy cows/herds. The 196 farmers who had reported the selected dead cows were asked to participate in a questionnaire survey that was part of a larger study on dairy cow mortality [2] (data from the 2002 questionnaire survey has previously been presented in [2]). The number of farmers participating in each questionnaire survey was determined based on a sample size calculation that is presented in [2]. The sampling protocol guaranteed that an equal number of cows, which had died every day of the week, were sampled. Only cows of dairy breeds and currently in commercial milk-producing herds were included. Cows from herds where all cows were euthanized because of occurrence of bovine spongiform encephalopathy (BSE) were not included (until July 2008, a total of 14 Danish herds have been infected with BSE [4]). Cows from educational or experimental herds were also excluded from the study. Immediately following the daily sampling protocol, a letter of introduction was sent to the farmers. The letter explained the background and purpose of the study and guaranteed confidentiality. To minimize recall bias, farmers were contacted by telephone 2 to 7 days following the mailing of the letter. If the farmer was not reached by phone, we attempted calling each subsequent day up to a maximum of four days. The farmer was censored if we failed to make contact. If farmers were excluded from the study an additional dead cow was identified from the database and the owner contacted in the same manner as described above. The resulting data set contained 196 dead cows and the corresponding interview from the 196 owners of these animals in each of the two years sampled (in total 392 farmers interviews).

The farmers were asked whether the cow died unassisted or was euthanized (closed question). Additionally, the farmers were asked their opinion regarding changes in their practice concerning euthanasia over the past 5 years using a closed question. The possible answers were: 1) I have lowered my threshold for euthanasia compared to 5 years ago, 2) my threshold is unchanged compared to 5 years ago, and 3) my threshold has increased compared to 5 years ago. If the farmer had started his/her operation less than 5 years ago the question was classified as 'not relevant'.

In the 2006 questionnaire survey the new sample of farmers were interviewed in exactly the same manner as described above. Additionally, they were interviewed about the reasons behind possible changes in their practice concerning euthanasia using an open question. Trends in prices of live dairy cows and beef meat over time were compiled from public statistics and used in the discussion of possible changes in the practice concerning euthanasia.

## Results and discussion

**Figure 1:**
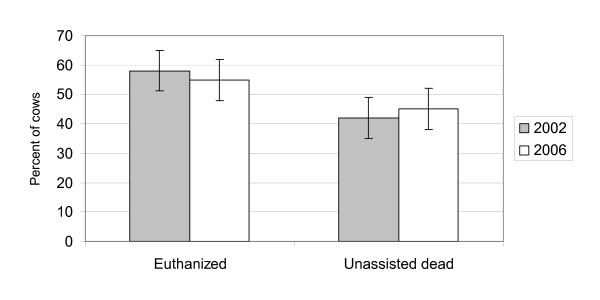
**Proportion of a random sample of 196 dead cows that were euthanized or died unassisted in 2002 and 2006, respectively**. 95% confidence intervals are indicated by vertical lines.

**Figure 2:**
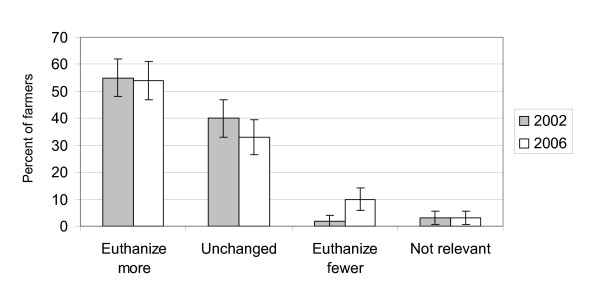
**The distribution of answers to a question regarding the farmers' practice concerning euthanasia of cows in 2002 and 2006, respectively, compared with 5 years earlier.** 95% confidence intervals are indicated by vertical lines.

The proportion of euthanized cows was approximately similar in the two surveys from 2002 and 2006 (Figure 1). Confidence intervals for the two years overlap and in fact the 'true' proportion of euthanized cows might be higher in 2006 than in 2002 and visa versa. In both surveys the farmers generally reported that their threshold for euthanasia had lowered compared to 5 years earlier (Figure 2). This was especially the case in the 2002 survey. Here 55% of the farmers reported a lower threshold and therefore reported euthanasia relatively more frequent (expressed as the percentage of cows in the herd euthanized per year) than 5 years earlier. Almost all the other farmers reported that they had the same threshold and therefore euthanized the same relative number of cows as 5 years earlier. In 2006, 54% reported a lower threshold and therefore performed euthanasia relatively more frequent than 5 years earlier, 33% reported unchanged threshold and performed euthanasia at the same level as previously and 10% reported higher threshold and euthanized relatively fewer cows than 5 years earlier. A total of 7 farmers participated in both the 2002 and the 2006 questionnaire survey. Recall bias regarding whether the cow died unassisted or was euthanized is unlikely due to the short time from the death of the cow to the interview. Recall bias regarding the changes in practice concerning euthanasia over time is more likely. Farmers might have difficulty remembering exactly how their policy regarding euthanasia was 5 years ago. However, our results regarding changes in euthanasia practice were relatively clear and recall bias most likely only affected our results to a minor degree. Additional evidence saying that the threshold for euthanasia has lowered during the years is not available at the moment. A number of technicalities prevent us from using information about health remarks from slaughterhouses for such an evaluation. Additional research is needed in this area.

**Figure 3:**
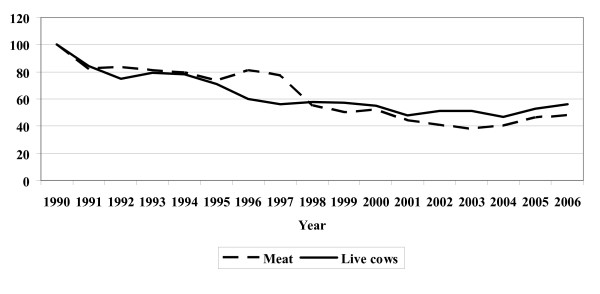
**Indexed prices of beef meat and live cows sold for dairy purposes in Denmark from 1990 to 2006 (1990 = index 100).** All prices are calculated in Danish kroner and adjusted for inflation. (Modified after [11-16]).

Interviews with the farmers in 2006 revealed a number of possible reasons (trends) behind the change in behaviour (Figure 2). Decreasing average profits per cow, decreasing value of the individual cow, increasing labour costs and increasing veterinary expenses during the last decade affected the farmer's decision-making concerning treatment versus euthanasia. Currently, the individual cow is not valuable to the farmer in the same way as 10 or 20 years ago [5,6]. Figure 3 illustrates the development in meat prices and prices of live cows from 1990 to 2006. It can be seen that prices have decreased steadily from 1990 to 2001–2003. Hereafter prices have increased again by approximately 20%. Additionally, the costs of treating a seriously ill cow have increased steadily. Consequently, the farmer's interest in intensive treatment of seriously ill cows has decreased and euthanasia has become an attractive alternative to treatment attempts. The increasing prices of meat and live cows during the last few years might explain the higher proportion of farmers having increased their threshold for euthanasia in 2006 compared to 5 years earlier compared with the situation in 2002 and 5 years before that.

Additionally, many farmers stated that changes in the practice concerning the veterinary inspection at the Danish slaughterhouses have affected their behaviour. A cow that was considered fit for transport a few years ago, is now often considered unfit for transport. If a farmer chooses to send such a cow for slaughter, the case may be considered as a violation of animal protection laws and consequently result in a fine to the farmer. To avoid this situation, many farmers therefore probably choose to euthanize cows whose fitness for transport is questionable. Some of the cows that are currently euthanized in the herds would probably have been sent to slaughter a few years ago. Besides a relatively new law against the slaughter of cows in the last tenth of pregnancy, these changes at the slaughterhouses are not caused by changes in any laws or regulations [7], but are most likely a result of a generally increased debate about and focus on the welfare of animals being transported for slaughter in Denmark in recent years [8]. This debate on animal welfare might have affected the awareness of veterinarians at the slaughterhouses and caused them to lower their threshold between what is acceptable and not when it comes to the transportation of animals.

We have no evidence indicating whether the number of seriously ill cows has increased during the last decade and therefore we are not able to quantify to what extent an increasing prevalence of euthanized cows might also have been affected by an increasing number of seriously ill cows. The number of recorded disease treatments in Danish dairy cows has, however, not changed significantly during the last 10–15 years [9]. It should be noted that the number of recorded disease treatments is not necessarily a good indicator of the 'true' disease status in the population. There are a number of steps from a cow being sick to a treatment record. If the farmer does not observe that the cow is sick, no treatment is initialised and hence no treatment is recorded. If the farmer observers that the cow is sick, he/she might for different reasons decide not to treat the cow [10]. Again, no treatment is recorded. And finally, if the farmer observes a sick cow and decides to treat her, the treatment might not be recorded correctly.

As a consequence of the findings from this study, as of the end of 2007, farmers are required to report to the Danish Cattle Database whether a dead cow died unassisted or was euthanized thus allowing differentiation between unassisted dead and euthanized cows in the future and evaluation of the welfare implications of possible changes in the proportion of euthanized cows.

## Conclusion

Other studies have shown that mortality risk in Danish dairy cows has increased significantly since 1990. Results from interviews with farmers, however, indicate that the threshold for euthanasia of cows among farmers has lowered. This situation might have a positive impact on animal welfare as more seriously ill cows are euthanized in the herds and not put through a period of suffering associated with disease and treatment or transport to a slaughterhouse in poor condition.

## Competing interests

The authors declare that they have no competing interests.

## Authors' contributions

PTT participated in the planning of the study, conducted the study, analysed the results and drafted the manuscript. JTS participated in the planning of the study and helped drafting the manuscript. Both authors read and approved the final manuscript.
